# Identification of cell and disease specific microRNAs in glomerular pathologies

**DOI:** 10.1111/jcmm.14270

**Published:** 2019-04-04

**Authors:** Janina Müller‐Deile, Jan Dannenberg, Peidi Liu, Johan Lorenzen, Jenny Nyström, Thomas Thum, Mario Schiffer

**Affiliations:** ^1^ Department of Medicine/Nephrology Friedrich–Alexander University Erlangen Erlangen Germany; ^2^ Department of Medicine/Nephrology Hannover Medical School Hannover Germany; ^3^ Department of Physiology Institute of Neuroscience and Physiology the Sahlgrenska Academy University of Gothenburg Gothenburg Sweden; ^4^ Department of Medicine/Nephrology University of Zurich Zurich Switzerland; ^5^ Institute of Molecular and Translational Therapeutic Strategies Hannover Medical School Hannover Germany; ^6^ REBIRTH Excellence Cluster Hannover Medical School Hannover Germany

**Keywords:** diagnostic marker, glomerular diseases, microRNA‐screening, renal cells, urine

## Abstract

MicroRNAs (miRs) are small non‐coding RNAs that regulate gene expression in physiological processes as well as in diseases. Currently miRs are already used to find novel mechanisms involved in diseases and in the future, they might serve as diagnostic markers. To identify miRs that play a role in glomerular diseases urinary miR‐screenings are a frequently used tool. However, miRs that are detected in the urine might simply be filtered from the blood stream and could have been produced anywhere in the body, so they might be completely unrelated to the diseases. We performed a combined miR‐screening in pooled urine samples from patients with different glomerular diseases as well as in cultured human podocytes, human mesangial cells, human glomerular endothelial cells and human tubular cells. The miR‐screening in renal cells was done in untreated conditions and after stimulation with TGF‐β. A merge of the detected regulated miRs led us to identify disease‐specific, cell type‐specific and cell stress‐induced miRs. Most miRs were down‐regulated following the stimulation with TGF‐β in all cell types. Up‐regulation of miRs after TGF‐β was cell type‐specific for most miRs. Furthermore, urinary miRs from patients with different glomerular diseases could be assigned to the different renal cell types. Most miRs were specifically regulated in one disease. Only miR‐155 was up‐regulated in all disease urines compared to control and therefore seems to be rather unspecific. In conclusion, a combined urinary and cell miR‐screening can improve the interpretation of screening results. These data are useful to identify novel miRs potentially involved in glomerular diseases.

## 1 INTRODUCTION

MicroRNAs (miRs) are small non‐coding RNAs that play an important role in gene regulation.^1^, ^2^, ^3^ In the nucleus primary miRs are transcribed by Ribonucleic acid (RNA) polymerases and then cleaved into double‐stranded miR precursors (Pre‐miRs) by RNase III enzyme (Drosha) and DiGeorge syndrome critical region 8 (DGCR8).^1^, ^2^ Pre‐miRs are exported into the cytoplasm where they are further cleaved into a guide strand and a passenger strand by the enzyme Dicer. Then the guide strand is loaded onto the RISC that binds to the 3′ untranslated region (3′UTR) of a target messenger RNA and inhibits RNA translation.^4^ Several miRs are enriched in human kidney and miRs seem to play a role in the glomerular homeostasis.^5^ Mice with podocyte‐specific alteration in miR‐expression by deletion of Dicer or Drosha display progressive glomerular damage with proteinuria and podocyte defects.^6^, ^7^ MiRs can be secreted in body fluids and therefore could possibly serve as biomarkers in various glomerular diseases. For example, patients with focal segmental glomerulosclerosis (FSGS) had 10‐times elevated concentrations of miR‐3d and miR‐10a in the urine compared to healthy controls.^8^ Urinary expression of miR‐200a, miR‐200b and miR‐429 were down‐regulated in patients with IgA glomerulonephritis (IgA‐GN) and this down‐regulation correlated with severity of the disease and rate of progression.^9^ MiRs offer some important advantages over other markers as they are stable and protected from endogenous RNase because of their small size and by packaging within exosomes.^10^, ^11^ However, although suggested as potential biomarkers the origin of miRs has rarely been defined and cell type specific miRs have not yet been reported in the kidney. In the past miR‐screenings in body fluids or tissue samples of patients with glomerular diseases were compared to healthy controls.^12^, ^13^, ^14^ By this approach, comparing samples of one disease to control samples makes the specificity of the findings elusive. In addition, it is not clear if the miRs are ‘bystanders’ or might originate from cells and tissues involved in the disease. Urinary miRs might be filtered from the blood, excreted by tubular cells or derived directly from glomerular cells affected by the disease process.

In this study, we described the advantages of a combined miR‐screening in urine as well as in cultured renal cells and explained different ways of data normalization and interpretation. We identified renal cell type‐specific miRs and miRs specifically regulated by TGF‐β in these cells. Hereby, we were able to investigate miRs in urines from patients with different glomerular diseases and could compare them to controls. In addition, we could assign the urinary miRs to the different renal cell types.

## 2 MATERIALS AND METHODS

### 2.1 Cell culture

Under permissive conditions at 33°C, podocytes proliferate. When cultured at 37°C, the SV40 T‐antigen was inactivated for cell differentiation. Culture medium for human podocytes was RPMI 1640 Medium (Roth, Karlsruhe, Germany) with 10% foetal calf serum (FCS; PAA Laboratories, Pasching, Australia), 1% Penicillin/Streptomycin and 0.1% Insulin. Human proximal tubular cells were cultured with renal epithelial cell media (Promocell, Baden‐Württemberg, Germany) with 5% FCS (PAA Laboratories, Pasching, Australia), 10 ng/mL recombinant human epidermal growth factor, 5 μg/mL recombinant human Insulin, 0.5 μg/mL Epinephrine, 36 ng/mL Hydrocortisone, 5 μg/mL Transferrin and 4 pg/mL Triiodo‐L‐thyronine. Human glomerular endothelial cells (Clonetech, Mountain View, CA) were cultured in endothelial cell basal media (EBM^™^‐2; CC‐3156, Lonza; Fisher). This medium was added with endothelial cell growth medium that contains 0.1% hEGF, 0.1% hydrocortison, 0.4% hFGF‐b, 0.1% VEGF, 0.1% R3‐IGF‐1, 0.1% Ascorbic Acid, 0.1% Heparin, 2% FBS and 0.1% GA. Human mesangial cells were cultured in Dulbecco's Modified Eagles's medium supplemented with 10% FCS and 1% Penicillin‐Streptomycin. Culture Conditions were 37°C and 5% CO_2_ air atmosphere. Cells were stimulated with 5 ng/mL human TGF‐β1 (Sigma Aldrich, Darmstadt, Germany) or high glucose (50 nmol/L). Cells were harvested 48 hours later with Quiazol for RNA isolation Urine sample preparation for miR‐screening. Morning urine was collected from healthy volunteers and from patients with biopsy‐proven glomerular diseases: FSGS, membranous glomerulonephritis (MGN), membranoproliferative glomerulonephritis (MPGN), diabetic nephropathy (DN), minimal change disease (MCD), preeclampsia (PREEC), haemolytic uraemic syndrome (HUS) and IgA‐glomerulonephritis (IgA‐GN). Ethical approval was obtained from Ethics Committee of the Hanover Medical School (#1709‐2013). In total 36 patients were included in the study. Urine samples (50 mL) were centrifuged at 75455 *g* for 15 minutes to pellet the cells and cellular debris. The cell‐free urine supernatant was stored at −80°C until miR‐screening analysis. Pooled urine samples from four patients per disease were used in the miR‐screening.

### 2.2 MiR‐isolation and miR‐screening

Purification of total RNAs including miRs from cultured renal cells and cell‐free urine from patients was done with miRNeasy Kit (QIAGEN, Venlo, Netherlands). QIAzol lysis reagent was added to the samples, mixed and incubated for 5 minutes. Five microlitres μL of 5 nmol/L Syn‐cel‐miR‐39 was added to each urinary sample to control for variations during preparation and later normalization for endogenous miRs. Chloroform was added to the samples, they were centrifuged and the upper phase containing RNA was transferred to a new collection tube. The RNA was then isolated with the help of RNeasy Mini spin columns and different buffers according to the manufactures’ instructions.

MiR‐screening in all cell types and in all urine samples was done with TaqMan^®^ Array Human MicroRNA Card Set v3.0 and Megaplex^™^ RT Primers Human Pool Set v3.0 (Life Technologis, Carlsbad, CA). The set enables quantitation of 754 human miRs and includes endogenous control for data normalization and one TaqMan^®^ MicroRNA Assay not related to human as a negative control. Pre‐amplification of miRs before the screening analysis was done with Megaplex^™^ PreAmp Primers, Human Pool Set v3.0 (Life Technologis) according to manufactures’ instructions.

### 2.3 Data analysis

We used the delta‐delta cycle threshold (CT) method to normalize our miR‐screening data and to generate fold changes in miR‐expression after TGF‐β stimulation and fold changes in miR‐expression in urine samples from patients with glomerular diseases compared to control.

Delta‐delta CT = delta CT (sample) – delta CT (reference) with delta CT (sample) = CT value for sample normalized to endogenous housekeeping gene and delta CT (reference) = CT value for calibrator normalized to endogenous housekeeping gene. The CT is defined as the number of cycles required for the fluorescent signal to cross the threshold (ie exceeds background level). CT levels are inversely proportional to the amount of target nucleic acid in the sample.

The maximum allowable CT value in our study was set to 35. MiR‐samples with bad intensity quality were excluded in the analysis. The normalization of the miR‐screening data of the different cell lines was done with the housekeeper U6 snRNA‐001973. Other like RNU48 or RNU44 was not used because they were regulated in our cell‐screening. This is in line with published data showing that these small‐nucleolar RNAs like RNU44, RNU48, RNU43 and RNU6B commonly used for miR‐normalization are regulated in tumours.^15^ The housekeeper for the miR‐analysis in urines was cel‐39 that was spiked into pooled urines of each disease before miR‐isolation. Normalized CT values were then transformed into relative quantity (RQ) value according to formula 2^−(delta‐delta CT)^. MiRs with RQ values >1.5 were considered up‐regulation and <0.5 were considered down‐regulation.

## 3 RESULTS

### 3.1 MiR‐screening setup

**Figure 1:**
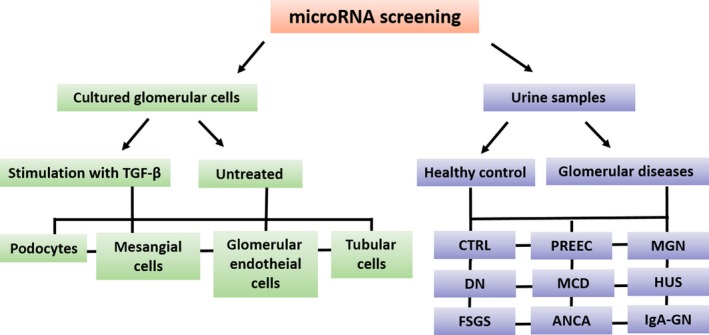
MiR‐screening in renal cell types and urines from patients with different glomerular diseases. Illustration of miR‐screening setup in cultured renal cell types and in urine samples of patients with different glomerular diseases. MiR‐screenings were done with TaqMan^®^ Array Human MicroRNA Card Set v3.0. Cells were left either untreated or stimulated with TGF‐β for 48 h. DN, diabetic nephropathy; FSGS, focal segmental glomerulosclerosis; HUS, haemolytic uraemic syndrome; IgA‐GN, IgA‐glomerulonephritis; MCD, minimal change disease; MGN, membranous glomerulonephritis; MPGN, membranoproliferative glomerulonephritis; PREEC, preeclampsia

We performed a Q‐PCR based miR‐screening (TaqMan^®^ Array Human MicroRNA Card Set v3.0) in cultured human podocytes, human glomerular endothelial cells, human mesangial cells and human proximal tubular cells in unstimulated conditions and after stimulation with TGF‐β. The same miR‐screening was performed in pooled urine samples from patients with FSGS, MGN, MPGN, DN, MCD, PREEC, HUS, IgA‐GN and healthy controls. The screening enabled the detection of 754 different human miRs. A schematic illustration of the miR‐screening is given in Figure 1. Patients’ characteristics are given in Table 1. Urine samples of two women and two men (except PREEC: urine samples of four women) with an active form of their disease were used for the miR‐screening.

**Table 1:** Characteristics of patients from the urinary miR‐screening

Disease	Age [years]	sex	serum creatinine [μmol/l]	UPC ratio [μg/ml]
MGN	81	w	83	5.474
MGN	65	w	103	1.617
MGN	59	m	127	11.352
MGN	30	m	194	10.270
FSGS	18	w	110	5.873
FSGS	32	w	146	19.562
FSGS	47	m	239	1.962
FSGS	42	m	273	5.500
MCD	44	w	60	2.614
MCD	42	w	42	3.660
MCD	25	m	99	6.413
MCD	14	m	73	2.986
DN	52	w	76	1.149
DN	41	w	76	265
DN	67	m	112	1.808
DN	41	m	146	8.920
PEEC	38	w	‐	5.431
PEEC	35	w	‐	4.834
PEEC	37	w	‐	1.371
PEEC	34	w	‐	1.852
ANCA	60	w	315	2.077
ANCA	50	w	206	608
ANCA	70	m	126	170
ANCA	64	m	150	1.853
IgA‐GN	51	w	78	5.100
IgA‐GN	12	w	42	169
IgA‐GN	44	m	167	2.525
IgA‐GN	49	m	112	862
HUS	75	w		
HUS	44	w		
HUS	58	m		
HUS	40	m		

DN, diabetic nephropathy; FSGS, focal segmental glomerulosclerosis; HUS, haemolytic uraemic syndrome; IgA‐GN, IgA‐glomerulonephritis; MCD, minimal change disease; MGN, membranous glomerulonephritis; MPGN, membranoproliferative glomerulonephritis; PREEC, preeclampsia.

### 3.2 Renal cell type‐specific miRs

By comparing the individual expression levels of each miR in the different cell types to the mean expression level of this miR in all cell types, we were able to detect cell type‐specific miRs (Table S1). Thereby, we could identify miR‐143‐3p as podocyte‐specific miR that was highly expressed in this cell type (fold change 36 in human podocytes compared to 0.37 in human glomerular endothelial cells, 0.27 in human mesangial cells and 0.25 in human tubular cells). In previous experiments we have already documented the importance of miR‐143‐3p for podocyte function and ultrastructure.^16^ Examples for glomerular endothelial cell and amesangial cell‐specific miRs are miR‐126 and miR‐206, respectively. MiR‐9 was exclusively expressed in tubular cells.

**Figure 2:**
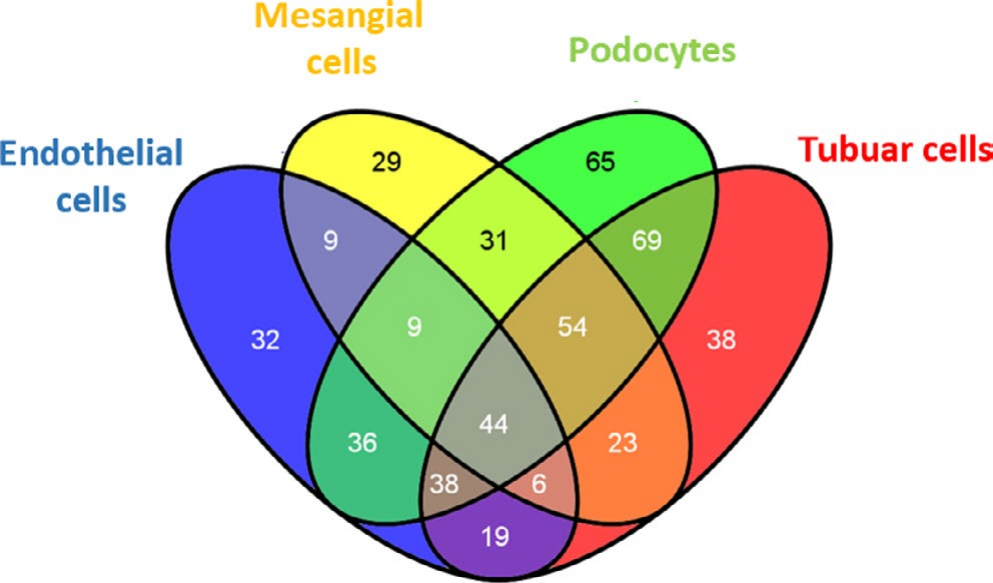
MiR‐screening in different cultured renal cell types. Venn diagram shows miRs found in different un‐stimulated cultured renal cells with the TaqMan^®^ Array based miR‐screening. The different colours in the Venn result from overlapping miRs in the different cell types. Number of miRs only found in one cell type (fields at the extreme end of the venn diagrams) or expressed in different cell types are indicated in the corresponding fields

We examined the overlap of miR‐expression in the different cell types. Twenty‐nine different miRs were only expressed in cultured human mesangial cells, 32 miRs were specific for human glomerular endothelial cells, 65 miRs were only detectable in human podocytes and 38 miRs were specific for proximal tubular cells (Table 2). Nineteen different miRs could be detected in all four renal cell types (Figure 2).

**Table 2:** Cell type‐specific miRs in cultured human glomerular endothelial cell, mesangial cells, podocytes and tubular cells

Endothelial cells	Mesangial cells	Podocytes		Tubular cells
hsa‐miR‐1197	hsa‐miR‐1269	hsa‐miR‐106b#	hsa‐miR‐499‐3p	hsa‐miR‐101#
hsa‐miR‐1291	hsa‐miR‐129#	hsa‐miR‐10a	hsa‐miR‐502	hsa‐miR‐1262
hsa‐miR‐140‐3p	hsa‐miR‐129	hsa‐miR‐10b	hsa‐miR‐505#	hsa‐miR‐1278
hsa‐miR‐192	hsa‐miR‐138‐2#	hsa‐miR‐1180	hsa‐miR‐517a	hsa‐miR‐139‐3p
hsa‐miR‐31#	hsa‐miR‐141	hsa‐miR‐1201	hsa‐miR‐517c	hsa‐miR‐16‐2#
hsa‐miR‐31	hsa‐miR‐196b	hsa‐miR‐1292	hsa‐miR‐518e	hsa‐miR‐182
hsa‐miR‐337‐3p	hsa‐miR‐202	hsa‐miR‐1293	hsa‐miR‐548b	hsa‐miR‐182#
hsa‐miR‐337‐5p	hsa‐miR‐320	hsa‐miR‐145#	hsa‐miR‐548c‐5p	hsa‐miR‐183#
hsa‐miR‐339‐3p	hsa‐miR‐326	hsa‐miR‐146b	hsa‐miR‐548H	hsa‐miR‐20b#
hsa‐miR‐34a#	hsa‐miR‐449	hsa‐miR‐198	hsa‐miR‐582‐5p	hsa‐miR‐296‐3p
hsa‐miR‐411	hsa‐miR‐509‐5p	hsa‐miR‐200a	hsa‐miR‐584	hsa‐miR‐346
hsa‐miR‐431	hsa‐miR‐517#	hsa‐miR‐23a	hsa‐miR‐589	hsa‐miR‐363
hsa‐miR‐433	hsa‐miR‐520f	hsa‐miR‐26a‐2#	hsa‐miR‐604	hsa‐miR‐363#
hsa‐miR‐485‐3p	hsa‐miR‐572	hsa‐miR‐302b	hsa‐miR‐615‐5p	hsa‐miR‐452
hsa‐miR‐539	hsa‐miR‐630	hsa‐miR‐361	hsa‐miR‐629	hsa‐miR‐132#
hsa‐miR‐624	hsa‐miR‐657	hsa‐miR‐130b#	hsa‐miR‐369‐5p	hsa‐miR‐152
hsa‐miR‐656	hsa‐miR‐674	hsa‐miR‐27b#	hsa‐miR‐372	hsa‐miR‐505
hsa‐miR‐770‐5p	hsa‐miR‐1263	hsa‐miR‐144	hsa‐miR‐18a#	hsa‐miR‐508
hsa‐miR‐938	hsa‐miR‐181a‐2#	hsa‐miR‐26a	hsa‐miR‐566	hsa‐miR‐520c‐3p
hsa‐miR‐941	hsa‐miR‐494	hsa‐miR‐34b	hsa‐miR‐582‐3p	hsa‐miR‐551b#
hsa‐miR‐942	hsa‐miR‐509‐3‐5p	hsa‐miR‐1228#	hsa‐miR‐641	hsa‐miR‐554
hsa‐miR‐130a#	hsa‐miR‐556‐5p	hsa‐miR‐429	hsa‐miR‐662	hsa‐miR‐561
hsa‐miR‐216a	hsa‐miR‐593	hsa‐miR‐432	hsa‐miR‐663B	hsa‐miR‐575
hsa‐miR‐543	RNU48	hsa‐miR‐449b	hsa‐miR‐720	hsa‐miR‐623
hsa‐miR‐574‐3p	hsa‐miR‐191	hsa‐miR‐450b‐3p	hsa‐miR‐744#	hsa‐miR‐627
hsa‐miR‐629	hsa‐miR‐380‐3p	hsa‐miR‐483‐3p	hsa‐miR‐758	hsa‐miR‐646
hsa‐miR‐668	hsa‐miR‐672	hsa‐miR‐483‐5p	hsa‐miR‐765	hsa‐miR‐649
hsa‐miR‐130a#	hsa‐miR‐1274A	hsa‐miR‐488	hsa‐miR‐769‐5p	hsa‐miR‐651
hsa‐miR‐216a	hsa‐miR‐220	hsa‐miR‐489	hsa‐miR‐872	hsa‐miR‐664
hsa‐miR‐543			hsa‐miR‐873	hsa‐miR‐92a‐2#
hsa‐miR‐574‐3p			hsa‐miR‐874	hsa‐miR‐92b#
hsa‐miR‐629			hsa‐miR‐876‐3p	hsa‐miR‐943
hsa‐miR‐668			hsa‐miR‐876‐5p	mmu‐miR‐374‐5p
			hsa‐miR‐887	hsa‐miR‐491‐3p
			hsa‐miR‐9#	hsa‐miR‐500
				hsa‐miR‐197
				hsa‐miR‐886‐5p
				hsa‐miR‐93#

Next, we compared the expression level of each miR and cell type to the global mean of all 754 miRs from the screening to categorize them in rather high (fold change >10) or low (fold change <0.5), expressed (Table S2). This analysis revealed that miR‐126, miR‐126#, miR‐531 and mir‐346 were not only glomerular endothelial cell‐specific but also highly expressed in this cell type (fold change >10 compared to the global mean of all miRs). MiR‐106a# and miR‐302a were highly expressed only in mesangial cells. Furthermore, miR‐200b, miR‐1225, miR‐221, miR‐1267 and miR‐331were specific for podocytes and expressed more than 10‐fold in this cell type compared to the global mean. Four different miRs were rather high expressed and specific for tubular cells: miR‐1305, miR‐499‐3p, let‐7b and miR‐454 (Table S2).

A third way of analysis is comparing the mean miR‐expression levels of all cell types to the global mean of miR‐expression level of all miRs, Again, we looked for miRs that where only expressed more than 10‐fold in one cell type. This method gave rather similar but different results than the data analysis above. MiR‐126, miR‐581, miR‐1274A and miR‐126# were endothelial cell‐specific whereas miR‐106a#, miR‐484 and let 7b were specific for mesangial cell. For podocytes the same miRs as in the analysis above came up: miR‐200b, miR‐1225, miR‐221, miR‐1267 and miR‐331. MiR‐1305, miR‐520b and miR‐486 were only up‐regulated in tubular cells more than 10‐fold (Table S3).

### 3.3 MiRs regulated by TGF‐β in cultured renal cells

**Figure 3:**
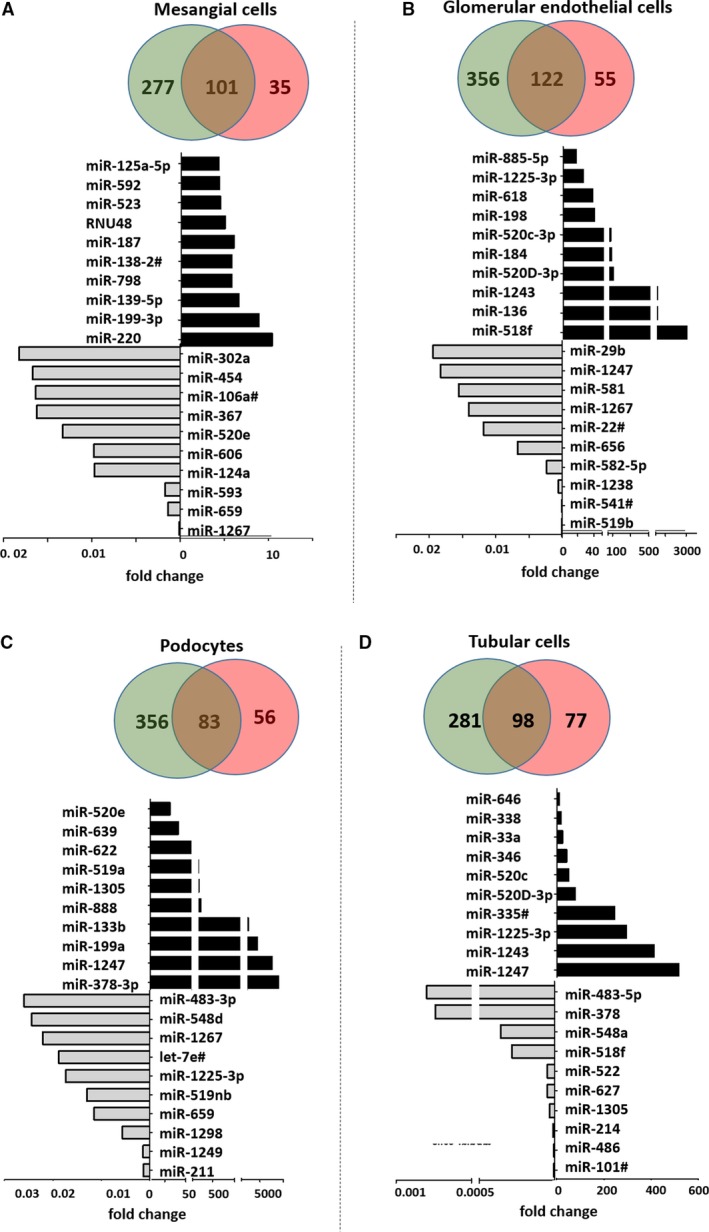
MiRs regulated by TGF‐β in different cultured renal cell types. Venn diagrams depict the number of miRs up‐regulated (fold change >1.5, red), down‐regulated (fold change <0.5, green) or unregulated (fold change >0.5 and <1.5, overlap) after TGF‐β in cultured human mesangial cells (A), human glomerular endothelial cells (B), human podocytes (C) and human tubular cells (D). Charts give the fold change in miR‐expression of the top 10 up‐regulated (black bars) and top 10 down‐regulated miRs after stimulation with TGF‐β compared to untreated condition

To identify cell stress inducible miRs we compared miR‐profiles from cultured renal cell lines before and after stimulation with TGF‐β. We generated fold changes in miR‐expression levels after TGF‐β stimulation (Table S4) and looked at the context cell type‐specific up‐ or down‐regulation of miRs. Figure 3A‐D gives the number of miRs upregulated (fold change >1.5, red), down‐regulated (fold change <0.5, green) or unregulated (fold change >0.5 and <1.5, overlap) after stimulation with TGF‐β in human mesangial cells (Figure 3A), human glomerular endothelial cells (Figure 3B), human podocytes (Figure 3C) and human tubular cells (Figure 3D).

Of note, most miRs were down‐regulated following the stimulation with TGF‐β in all cell types. Up‐regulation of miR‐expression after stimulation with TGF‐β was most prominent in cultured glomerular endothelial cells and podocytes. Moreover, up‐regulation of miRs after TGF‐β was cell type‐specific for most miRs. For example, miR‐378a‐3p was specifically up‐regulated in cultured human podocytes. We previously described the importance of miR‐378a‐3p for glomerular filter function and ultrastructure.^17^

Interestingly, the regulation of some miRs was concordant for more than one cell type. For example, miR‐199a‐3p was up‐regulated in both mesangial cells and podocytes. MiR‐1247 was up‐regulated not only in podocytes but also in tubular cells. MiR‐1243, miR‐1225‐3p, miR‐520D‐3p and miR‐520c‐3p were induced after stimulation with TGF‐β in glomerular endothelial cells as well as tubular cells. This suggests that these cell types regulate common pathways. It is striking that we found no miR significantly up‐regulated in more than two cell types, indicating that the miR‐regulation after TGF‐β is clearly cell type‐specific.

### 3.4 MiRs in cell‐free urines from patients with different glomerular diseases compared to control

**Figure 4:**
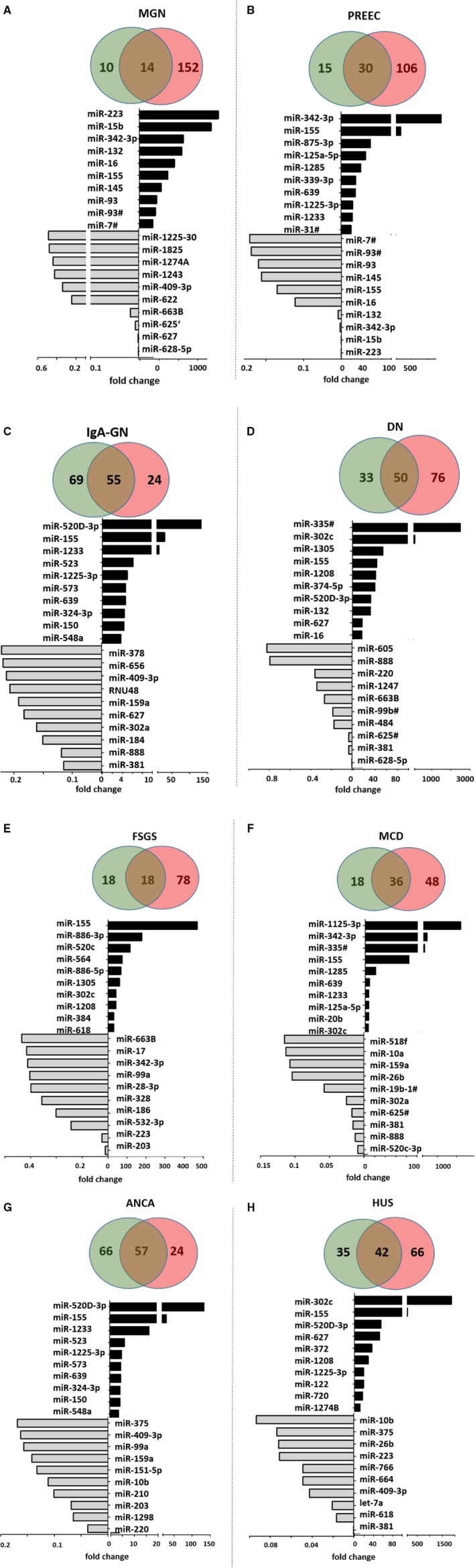
MiRs expressed in urines from patients with different glomerular diseases compared to control. Venn diagrams give the number of miRs up‐regulated (fold change >1.5, red), down‐regulated (fold change <0.5, green) or un‐regulated (fold change >0.5 and <1.5, overlap) in pooled urine samples from patients with MGN (A), PREEC (B), IgA‐GN (C), DN (D), FSGS (E), MCD (F), ANCA (G) and HUS (H) compared to pooled urines from healthy controls. Charts give the fold change in miR‐expression of the top 10 up‐regulated (black bars) and top 10 down‐regulated miRs in urines samples from patients with glomerular diseases compared to control. Abbreviations: DN, diabetic nephropathy; FSGS, focal segmental glomerulosclerosis; HUS, haemolytic uraemic syndrome; IgA‐GN, IgA‐glomerulonephritis; MCD, minimal change disease; MGN, membranous glomerulonephritis; MPGN, membranoproliferative glomerulonephritis; PREEC, preeclampsia

Next, we examined in a screening experiment urinary miR‐profiles from pooled urine samples from patients with different glomerular diseases and compared them to those from healthy controls (Table S5). Interestingly, except for ANCA and IgA‐GN, most miRs were higher expressed in disease urines compared to controls. Figure 4 gives the number of miRs up‐regulated (fold change >1.5, red), down‐regulated (fold change <0.5, green) or unregulated (fold change >0.5 and <1.5, overlap) in MGN (Figure 4A), PREEC (Figure 4B), IgA‐GN (Figure 4C), DN (Figure 4D), FSGS (Figure 4E), MCD (Figure 4F), ANCA (Figure 4G) and HUS (Figure 4H).

The 10 top up‐regulated and 10 top down‐regulated miRs in the urines from patients compared to healthy controls are depicted in the charts of Figure 4A‐H. Only one miR was up‐regulated in all disease urines compared to control. This was miR‐155.

MiR‐155 also caught our attention in the miR‐screening in the cells. It was much higher expressed in glomerular endothelial cells compared to other cell types (fold change 9.0, Table S1). It consistently was detected in high fold change in glomerular endothelial cells (miR‐expression analysis with the global mean of all miRs and miR‐expression analysis with the local mean of all miRs; Tables S2 and S3). After stimulation with TGF‐β miR‐155 was further up‐regulated in glomerular endothelial cells (fold change 5.4, Table S4).

We were able to identify disease‐specific miRs that were only detectable in one glomerular disease and not present in controls. These disease‐specific miRs were let‐7 g for MGN, miR‐99b# for PREEC, miR‐603 for FSGS and miR‐590 for ANCA.

### 3.5 Assigning urinary miRs to the different renal cell types

**Figure 5:**
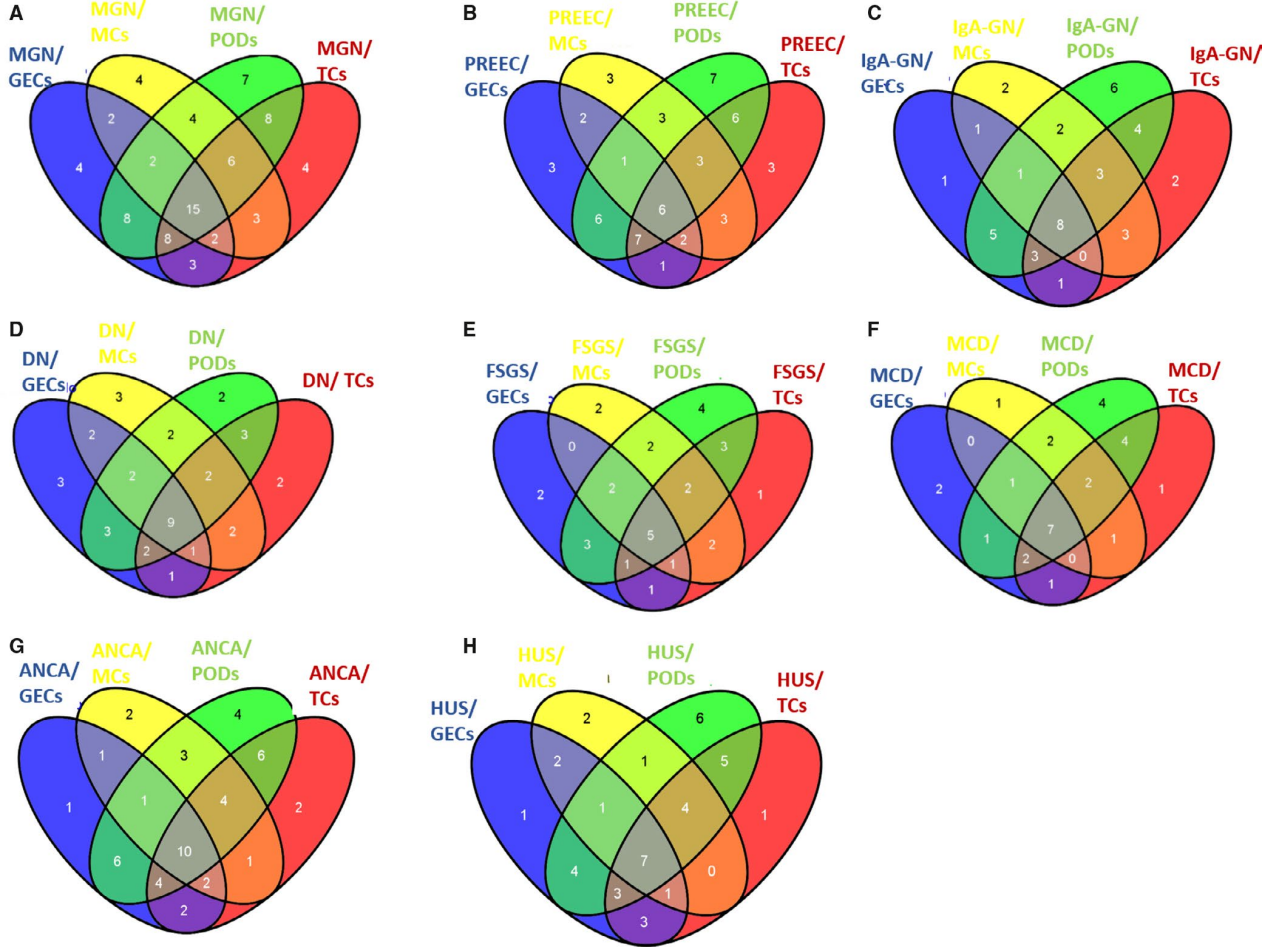
Merged results from urinary miRs of patients with different glomerular diseases and the renal cell types. Venn diagram depicts the miRs expressed in urine samples from patients with MGN (A), PREEC (B), IgA‐GN (C), DN (D), FSGS (E), MCD (F), ANCA (G) and HUS (H) to GECs, MCs, PODs and TCs. The different colours in the venn result from overlapping miRs in the different cell types. Number of miRs only found in one cell type (fields at the extreme end of the venn diagrams) or expressed in different cell types are indicated in the corresponding fields. Abbreviations: DN, diabetic nephropathy; GECS, glomerular endothelial cells; FSGS, focal segmental glomerulosclerosis; HUS, haemolytic uraemic syndrome; IgA‐GN, IgA‐glomerulonephritis; MCD, minimal change disease; MGN, membranous glomerulonephritis; MPGN, membranoproliferative glomerulonephritis; MCS, mesangial cells; PEEC, preeclampsia; PODS, podocytes; TCS, tubular cells

By combining the cell and urinary miR‐screenings, we could assign the miRs found in the urines from patients with glomerular diseases to the miRs identified in TGF‐β stressed cultured renal cells (Figure 5A‐H). The combination of cell and urine miR‐screenings enabled us to identify cell type‐specific miRs in the different glomerular diseases (Table 3 and Figure 5A‐H).

**Table 3:** Cell type‐specific miRs in the different glomerular diseases

**MGN + ENDO**	**MGN + ENDO**	**MGN + PODOS**	**MGN + TUBULUS**
hsa‐miR‐192‐000491	hsa‐miR‐1274A‐002883	hsa‐miR‐10a‐000387	hsa‐miR‐152‐000475
hsa‐miR‐200c‐002300	hsa‐miR‐141‐000463	hsa‐miR‐10b‐002218	hsa‐miR‐197‐000497
hsa‐miR‐31‐002279	hsa‐miR‐191‐002299	hsa‐miR‐146b‐001097	hsa‐miR‐886‐5p‐002193
hsa‐miR‐574‐3p‐002349	hsa‐miR‐320‐002277	hsa‐miR‐200a‐000502	hsa‐miR‐93#‐002139
		hsa‐miR‐26a‐000405	hsa‐miR‐99b‐000436
		hsa‐miR‐429‐001024	
		hsa‐miR‐720‐002895	
		hsa‐miR‐99b‐000436	
**FSGS + ENDO**	**FSGS + MESANG**	**FSGS + PODOS**	**FSGS + TUBULUS**
hsa‐miR‐200c‐002300	hsa‐miR‐141‐000463	hsa‐miR‐10a‐000387	hsa‐miR‐886‐5p‐002193
hsa‐miR‐31‐002279	hsa‐miR‐320‐002277	hsa‐miR‐146b‐001097	
		hsa‐miR‐26a‐000405	
		hsa‐miR‐720‐002895	
**IgA‐GN + ENDO**	**IgA‐GN + MESANG**	**IgA‐GN + PODOS**	**IgA‐GN + TUBULUS**
hsa‐miR‐200c‐002300	hsa‐miR‐1274A‐002883	hsa‐miR‐10a‐000387	hsa‐miR‐197‐000497
	hsa‐miR‐141‐000463	hsa‐miR‐10b‐002218	hsa‐miR‐93#‐002139
		hsa‐miR‐200a‐000502	
		hsa‐miR‐26a‐000405	
		hsa‐miR‐489‐002358	
		hsa‐miR‐720‐002895	
**DN + ENDO**	**DN + MESANG**	**DN + PODOS**	**DN + TUBULUS**
hsa‐miR‐200c‐002300	hsa‐miR‐1274A‐002883	hsa‐miR‐26a‐000405	hsa‐miR‐152‐000475
hsa‐miR‐31‐002279	hsa‐miR‐191‐002299	hsa‐miR‐720‐002895	hsa‐miR‐93#‐002139
hsa‐miR‐574‐3p‐002349	hsa‐miR‐320‐002277		
**MCD + ENDO**	**MCD + MESANG**	**MCD + PODOS**	**MCD + TUBULUS**
hsa‐miR‐200c‐002300	hsa‐miR‐191‐002299	hsa‐miR‐10a‐000387	hsa‐miR‐93#‐002139
hsa‐miR‐574‐3p‐002349		hsa‐miR‐10b‐002218	
		hsa‐miR‐146b‐001097	
		hsa‐miR‐720‐002895	
			
**HUS + ENDO**	**HUS + MESANG**	**HUS + PODOS**	**HUS + TUBULUS**
hsa‐miR‐31‐002279	hsa‐miR‐1274A‐002883	hsa‐miR‐10a‐000387	hsa‐miR‐886‐5p‐002193
	hsa‐miR‐191‐00229	hsa‐miR‐10b‐002218	
		hsa‐miR‐200a‐000502	
		hsa‐miR‐26a‐000405	
		hsa‐miR‐489‐002358	
		hsa‐miR‐720‐002895	
**PREE + ENDO**	**PREE + MESANG**	**PREE + PODOS**	**PREE + TUBULUS**
hsa‐miR‐200c‐002300	hsa‐miR‐1274A‐002883	hsa‐miR‐10a‐000387	hsa‐miR‐152‐000475
hsa‐miR‐31‐002279	hsa‐miR‐191‐002299	hsa‐miR‐10b‐002218	hsa‐miR‐197‐000497
hsa‐miR‐574‐3p‐002349	hsa‐miR‐320‐002277	hsa‐miR‐146b‐001097	hsa‐miR‐93#‐002139
		hsa‐miR‐26a‐000405	
		hsa‐miR‐429‐001024	
		hsa‐miR‐489‐002358	
		hsa‐miR‐720‐002895	
**ANCA + ENDO**	**ANCA + MESAG**	**ANCA + PODO**	**ANCA + TUBULUS**
hsa‐miR‐192‐000491	hsa‐miR‐1274A‐002883	hsa‐miR‐200a‐000502	hsa‐miR‐197‐000497
	hsa‐miR‐320‐002277	hsa‐miR‐26a‐000405	hsa‐miR‐93#‐002139
		hsa‐miR‐429‐001024	
		hsa‐miR‐489‐002358	

**Figure 6:**
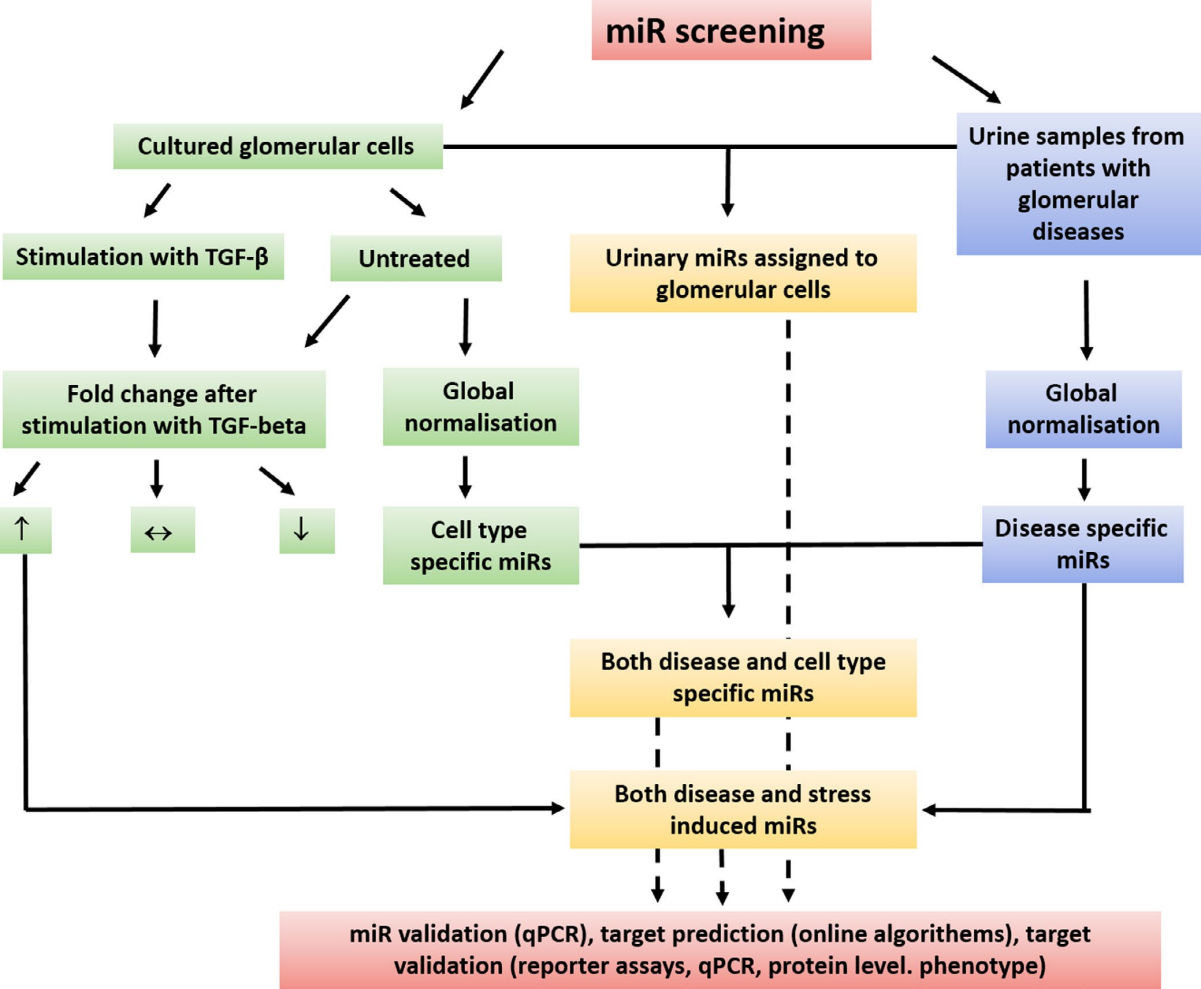
MiR‐screening analysis. Work flow of miR‐screening analysis

## 4 DISCUSSION

It is estimated that 60% of the total human proteome is regulated by about 2000 known miRs.^18^ MiRs also seem to play an important role in gene regulation in disease processes.

Therefore, miR‐screenings are novel tools to find diagnostic markers or even therapeutic targets in glomerular diseases. In the past, miR‐screenings were predominantly performed in serum or urine from patients. However, by analysing miRs in body fluids only (Table S5), the origin of the miRs as well as their pathophysiological role in disease remains elusive. Urinary miRs might be filtered or excreted by the kidney. Alternatively they might be directly derived from renal cells during the disease process.

We performed three different miR‐screenings with 745 different miRs: One in different renal cell types under normal culture condition, one in cultured renal cell types after stimulation with TGF‐β and another in urine samples from patients with different glomerular diseases. These data are the basis for different ways of analysis depending on the biological question to be answered.

Most studies on urinary miRs used the urine sediment obtained after low‐speed centrifugation. However, a large number of low‐quality and degraded RNA was recently detected in the urinary cell pellet.^19^ Therefore, we decided to use the cell‐free supernatants of pooled patient urines in our miR‐screening. We first wanted to identify potentially cell type‐specific miRs. We could find miR‐126 as one of the glomerular endothelial cell‐specific miRs in our screening. Well in line with this finding, miR‐126 was already described as an endothelial cell‐specific miR that governs vascular integrity in other tissue.^20^, ^21^, ^22^, ^23^

MiR‐143‐3p was predominantly expressed in podocytes in our miR‐screening. We have confirmed the importance of this miR for the maintenance of a functional glomerular filtration barrier in the zebrafish model.^16^ MiR‐30 family members were also highly expressed in our cultured human podocytes. In line with this, a role of the miR‐30 family in podocyte homeostasis was previously described in mice.^24^

The comparison of cellular miR‐profiles before and after stimulation with TGF‐β revealed cell stress‐induced miRs. Most miRs up‐regulated after TGF‐β were cell type‐specific and none was regulated in more than two renal cell types. Some cell type‐specific regulations were consistent with previous findings. For example, miR‐143‐3p and miR‐378a‐3p are known to be regulated by TGF‐β in non‐renal cells.^25^, ^26^ Both miRs were also up‐regulated in cultured human podocytes after stimulation with TGF‐β in our miR‐screening. Circulating miR‐210 predicts survival in critically ill patients with acute kidney injury.^27^ Interestingly, miR‐210 was up‐regulated after TGF‐β in human mesangial cells in our study. MiR‐199a‐3p was up‐regulated in human mesangial cells and podocytes after stimulation with TGF‐β. A known target of miR‐199a‐3p is versican also produced by podocytes and mesangial cells.^28^, ^29^

Most miRs were down‐regulated following the stimulation with TGF‐β in our miR‐screening in cultured human renal cell lines. TGF‐β is associated with the increase in many pro‐fibrotic and inflammatory markers. MiRs down‐regulation after TGF‐β might contribute to this increase in target expression levels. For example depletion of renal miR‐196a/b by miR‐196a/b antagomirs substantially aggravated unilateral ureteral obstruction‐induced renal fibrosis.^30^ During renal injury, reduction in miR‐29a and miR‐29b enhances collagen expression^31^ and down‐regulation of miR‐200a expression promotes TGF‐dependent epithelial‐to‐mesenchymal transition.^32^

The miRs down‐regulated in our cultured renal cell lines after stimulation with TGF‐β may give cues to look for interesting up‐regulated targets to prove TGF‐β mediated fibroses and inflammation regulated through miRs. Elevating miRs that are decreased by TGF‐β by miR‐mimics might have therapeutically potential.

Regarding the urinary miR‐screening, most miRs were higher expressed in patients with glomerular diseases compared to healthy controls. This might be due to the increased leakiness of the glomerular filtration barrier in glomerular disease that also allows more micro particles and RNA‐binding proteins associated with miRs to pass.^33^ Recently, urinary miR‐21, miR‐200c and miR‐423 have been identified as sensitive indicators of kidney injury. MiR‐21 was also described to inhibit pathophysiological pathways in DN.^34^

Nevertheless, the origin of these urinary miRs is unknown.^35^ In our screening, miR‐21 was expressed in cultured human mesangial cells and podocytes and was detectable as up‐regulated in urines from patients with IgA‐GN, FSGS, MCD, MGN, PREEC and DN.

In animal models of kidney injury, miR‐21 expression was found to be increased as well. However, its function remains controversial, because it has been implicated in promotion as well as protection from tubule‐interstitial and glomerular injury.^36^, ^37^, ^38^, ^39^

MiR‐21 was described to ameliorate TGF‐β and hyperglycemia‐induced glomerular injury through repression of pro‐apoptotic signals.^34^ In contrast to this, murine models of tubule‐interstitial kidney injury demonstrated that miR‐21 contributes to fibrogenesis and epithelial injury.^36^ In line with these findings, miR‐21 antagonism rescued mesangial expansion, interstitial fibrosis, macrophage infiltration, podocyte loss, albuminuria and fibrotic‐ and inflammatory gene expression in mice with diabetic nephropathy.^40^

In another study of diabetic kidney disease, miR‐21 enhanced high glucose‐induced TOR complex 1 activity, resulting in renal cell hypertrophy and fibronectin expression.^39^

Our findings and data from the literature suggest that miR‐21 might have diverse functions in different glomerular disease contexts and its regulation might be much more complex than initially suggested.

MiR‐378a‐3p was found in urines samples from patients with MGN, FSGS and MCD and up‐regulated in cultured human podocytes in response to TGF‐β treatment. In a previous study we could confirm the importance of miR‐378a‐3p for glomerular function as it is a regulator of the glomerular matrix protein nephronectin.^17^ Thus, our data confirm that urinary miRs seem to be markers for renal injury.

MiR‐155 was the only miR that was up‐regulated in all disease urines compared to control. In all different analysis strategies in cells including the analysis after TGF‐β‐stimulation, miR‐155 was highly up‐regulated in glomerular endothelial cells. TGF‐β‐regulation of miR‐155 has been described before.^41^ Well in line with our observation miR‐155 was previously described by others to play an important role in endothelial cell activation.^42^, ^43^

Patients with diabetic nephropathy displayed reduced levels of serum miR‐31. Moreover, miR‐31 levels were positively correlated with leucocyte rolling velocity and negatively associated to leucocyte adhesion, TNFα, IL‐6 and ICAM‐1 levels.^44^ We did not found miR‐31 among the top 10 miRs down‐regulated in diabetic nephropathy compared to control in our miR‐screening. This highlights the importance of the type of bio‐fluid investigated in miR‐studies.

Overexpression of miR‐370 was shown to promote mesangial cell proliferation and extracellular matrix accumulation by suppressing CNPY1 in a rat model of diabetic nephropathy. However, this miR was not among the top 10 miRs regulated in human renal cells or urines from patients. This indicated that a species‐specific miR‐analysis seems to be important as there might be differences in miR‐expression in humans and rats.^45^

Combining the cellular and urinary miR‐screening, we were able to define disease‐specific and cell type‐specific miR‐profiles and could assign the urinary miRs to the different renal cell types. To our knowledge this is the first study merging biological samples from patients with results from unstressed and stressed cultured cells to identify biological important miRs. Even though our screening aimed to identify miRs relevant in the pathology we confirmed several published previous observations by us and others. A limitation of our study is that the screening results have to be confirmed in independent experiments or larger patient cohorts. Nevertheless our approach uses a unique comparison in eight different disease groups and all corresponding glomerular cell types and is therefore a first approach to identify novel miRs potentially involved in the pathophysiology of glomerular diseases.

## CONFLICT OF INTEREST

None.
